# Reflection Symmetry Detection in Earth Observation Data

**DOI:** 10.3390/s23177426

**Published:** 2023-08-25

**Authors:** David Podgorelec, Luka Lukač, Borut Žalik

**Affiliations:** Faculty of Electrical Engineering and Computer Science, University of Maribor, Koroška cesta 46, SI-2000 Maribor, Slovenia; luka.lukac@um.si (L.L.); borut.zalik@um.si (B.Ž.)

**Keywords:** computer science, approximate symmetry, partial symmetry, local symmetry, point cloud, voxel, line segment

## Abstract

The paper presents a new algorithm for reflection symmetry detection, which is specialized to detect maximal symmetric patterns in an Earth observation (EO) dataset. First, we stress the particularities that make symmetry detection in EO data different from detection in other geometric sets. The EO data acquisition cannot provide exact pairs of symmetric elements and, therefore, the approximate symmetry must be addressed, which is accomplished by voxelization. Besides this, the EO data symmetric patterns in the top view usually contain the most useful information for further processing and, thus, it suffices to detect symmetries with vertical symmetry planes. The algorithm first extracts the so-called interesting voxels and then finds symmetric pairs of line segments, separately for each horizontal voxel slice. The results with the same symmetry plane are then merged, first in individual slices and then through all the slices. The detected maximal symmetric patterns represent the so-called partial symmetries, which can be further processed to identify global and local symmetries. LiDAR datasets of six urban and natural attractions in Slovenia of different scales and in different voxel resolutions were analyzed in this paper, demonstrating high detection speed and quality of solutions.

## 1. Introduction

An object or a system (a scene is used for both from here on) is symmetric if there is a transformation, such as a translation, rotation, reflection, or a combination of these, that maps it onto itself [1]. The concept of symmetry may be generalized to various abstractions, but a great majority of studies and demonstrations, including the proposed paper, are focused on the visual symmetry perception, which is characterized by the detection and interpretation of colors and distances (depth and size). Symmetry perception has become an integral part of the individual’s perceptual organization process, where individual regularities have been selected on the basis of their relevance [2]. Such phenomena have been recognized in studies of mating and food choice habits of many animal species, including birds, dolphins, apes, and bees [3]. In humans, this concept can be generalized to a broader perception of aesthetics, safety and stability, which explains its role in different fields of human activity, including arts, architecture, biology, medicine, mathematics, and various engineering disciplines [3,4]. Further studies have demonstrated that symmetry is important for the visual system by facilitating image segmentation as well as object analysis and representation [5,6]. However, in contrast to the natural, almost self-evident symmetry perception processes in living beings, computer-aided symmetry detection is anything but simple [4]. Algorithms of image processing and computational geometry, optimization methods, and domain knowledge must be carefully designed and integrated to provide relevant, accurate, and timely solutions.

Earth observation data are huge sets of spatial or spatiotemporal samples acquired from satellites [7,8], airborne platforms [9,10], and in situ measurements on land, in water, and in the atmosphere [11]. When processed appropriately, including feature extraction, spatial and/or color (or other acquired/measured variable) segmentation, and object modeling and analysis, EO data provide a wide variety of new insights into Earth’s processes and systems [12]. Furthermore, diverse EO datasets can be fused with each other [11] or with other geospatial (GIS) data [12]. Symmetry can also be a very useful feature in this context, due to its already explained role in visual perception. However, although the use of symmetries in EO data processing has been growing rapidly over the last two decades, it is still not reaching its potential. Practically all the applications we found in the literature, such as [13,14,15,16,17,18,19,20], assume that the symmetries are known in advance, either being detected by some general algorithm or manually identified. They do not introduce any specialized symmetry detection algorithms, which would benefit from the nature of the EO data. Symmetry is used in 3D architecture, street or network of streets reconstruction [13,14,15], detection of roads [16], and extraction of railway elements [17]. Individual tree delineation from EO data, particularly LiDAR point clouds, is a popular research topic, but symmetry usually plays only a marginal role in the methods, mostly incorporated in a form of assumptions to supplement other means of segmentation [18,19]. A symmetry quality measure was recently used to predict water level in one of the largest intermittent lakes in Europe from Sentinel-2 data [20]. All these solutions are narrowly focused to scenes consisting of clearly distinguishable components and, except the last three, with smooth surfaces and rigorously controlled noise.

Symmetry is thus a transformation f:S→S, where *S* is the set (the scene In or a part of it, where In refers to the input set) invariant under *f*. We will use the term symmetry a bit inconsistently for both *f* and *S*. The symmetry detection is aimed at answering two different questions:

1. Is a considered scene symmetric? This is the global symmetry detection problem, which is solved by trying to find a transformation, which concerns the whole scene, i.e., S=In.
2. Does the scene contain any (smaller) symmetric patterns? This is the partial symmetry detection, where the patterns with the property of being symmetric must be identified and extracted from the scene together with the attributes of the symmetry transformation, i.e., S⊆In.

The concept of the partial symmetry was used in this manner by Mitra et al. [21]. Other authors mostly consider a similar concept of the local symmetry instead, as discussed at the beginning of Section 2.

There are three basic symmetry types regarding the transformation function: reflection, rotational, and translational symmetry. We only consider detection of the reflection symmetry across a symmetry plane in 3D in a perpendicular direction (or across the symmetry axis in 2D). Note that other types of reflection symmetry exist where the mirroring transformation operates either across an arbitrary surface in 3D, across an arbitrary curve in 2D or 3D, or across a point in 1D, 2D, or 3D. The latter is known as central or radial symmetry. The mirroring direction need not be perpendicular.

In this paper, a new algorithm is presented, predominantly designed to detect partial reflection symmetries in Earth observation (EO) data. To the best of our knowledge, there are no symmetry detection algorithms tailored to the particularities of EO data. Existing applications of symmetries in EO data therefore typically use slower general algorithms or even manual symmetry labeling. The presented algorithm is required to detect multiple symmetries if they exist. The principle of maximality is employed to reduce their number. If there are two symmetries with the same symmetry plane [21], i.e., $f:S_{1}\to S_{1}$ and $f:S_{2}\to S_{2}$, and if $S_{1}\subset S_{2}$, then only $S_{2}$ is accepted as the solution. If, for example, a global symmetry is found as a special case of the partial symmetry, then none of its subsets are considered as a symmetry. With a small extension, the algorithm is also able to identify the so-called local symmetries. Section 1 is concluded with a survey of previous works on reflection symmetry detection, with emphasis on partial symmetry. A part of the survey addresses symmetry detection and applications in processing EO data. Section 2 stresses first the particularities that make symmetry detection in EO data different from detection in other geometric sets. The structure of the novel algorithm and the detailed descriptions of its individual parts are given afterwards. Section 3 demonstrates the high detection speed and quality of solutions through visualizations and analyses of LiDAR datasets of six urban and natural attractions in Slovenia. The results, some minor additional functionalities, limitations, possible improvements, extensions, and applications are discussed in the concluding Section 4.

### Related Works

In this subsection, we stress the main features and differences of the existing methods for global and local reflection symmetry detection, and their impact on the proposed approach. Mitra et al. [22] made an extensive survey, while some newer approaches were discussed in our related research work [4] recently.

The global reflection symmetry methods use a variety of different approaches to calculate the symmetry plane (or more of them) on the entire scene. Chen et al. [23] used the weighted primary component analysis (PCA) method to determine the initial symmetry plane, which was then refined iteratively by adjusting the weights. Schiebener et al. [24] determined the symmetry plane candidates by the RANSAC method. Combés et al. [25] and Ecins et al. [26] based their methods on an iterative nearest point search. Nagar and Raman [27] transformed the problem into the optimization problem on a smooth Riemannian product manifold. Hruda et al. [28] used a gradient-based optimization designed on their differential symmetry measure. Other concepts utilize edge detection proposed by Elawady et al. [29], multiple virtual viewpoints and their entropy distribution by Li et al. [30], heat diffusion by Sipiran et al. [31], and extended Gaussian images used by Sun and Sherrah [32] and Kakarala [33]. Some of the listed algorithms operate on 3D point clouds [23,24,25,26,27,28], some on triangular meshes [30,31,32], and some on both [33]. The method of Elawady et al. [29] is limited to raster images, which may be of interest for satellite images, aerial photographs, or orthophotos. In any case, from the point of view of the method introduced in this paper, the most interesting methods are those that process voxelized data. Korman et al. [34] proposed an algorithm with a scalable sampling rate, depending on the total variation of the shape. It also detects global rotational symmetries (such as [32]). Podolak et al. [35] first sampled the object’s surface by the Monte Carlo approach and embedded the samples into a voxel grid. All possible symmetry planes were then determined among the samples and evaluated by a symmetry measurement function. This method is particularly important because of its adaptation to local symmetry detection [36]. Unlike other described solutions, the method by Žalik et al. [4] rotates the candidate symmetry plane around the centroid and uses hierarchical space subdivision to identify good candidates.

Partial symmetry is not strictly and uniquely defined in the literature. Some authors use the term as a synonym for local symmetry, others for approximate global symmetry, and others for approximate symmetry in general. We use it as a superclass for both the global and local symmetry. The latter is sometimes handled by segmenting the scene into individual parts and then detecting global symmetry separately on each of them [24,26,31]. True local reflection symmetry detection methods were proposed in [21,36,37,38,39]. Simari et al. [37] and Calliere et al. [38] both proposed methods on triangular meshes. The former is based on the covariance matrix of weighted gravity centers, and the latter on the Hough transform. Mitra et al. [21] presented a powerful general method that handles global and local reflection and rotational symmetries in a point cloud. Curvature/Laplacian local shape descriptors are computed for each point, requiring that the points are topologically connected into the grid. The points with similar descriptors are paired. The pairs are clustered into symmetric patches, which are then connected in a graph. Hruda et al. [39] recently proposed the method, based on [21,28], which uses a simple PCA-based shape descriptor and does not require a grid. Speciale et al. [36] proposed two methods for voxelized scenes. The first one checks all the pairs of randomly selected voxels, as described in [35]. The second method marks the voxels with large enough gradients and curvatures as boundary voxels, pairs only these, and then additionally samples the pairs randomly to determine the candidates for symmetry planes.

Machine learning approaches are increasingly popular in this field as everywhere else, but they all require a rich training set and a relatively demanding and time-consuming learning process. Current solutions in 3D are limited to the global reflection symmetry either in point clouds [40,41] or voxel grids [42], while some solutions in raster images address local reflection symmetry as well [43].

## 2. The Method

The presented algorithm is predominantly designed to detect partial reflection symmetries in EO data. With some pre-processing and a few minor adjustments, other geospatial (GIS) data can also be processed, as we will explain in Section 4. An early version of the algorithm was presented in [44] already, but the method was upgraded with several improvements, speed-ups, and new functionalities since then. Much larger datasets with higher voxel resolutions, that were previously processed in tens of hours or even a few days, can be handled in few hours or even faster now. Before describing the structure of the method and detailing its building blocks, let us explain the concept of partial symmetry and the peculiarities of the EO data that had a significant impact on the design of the method.

**Figure 1:**
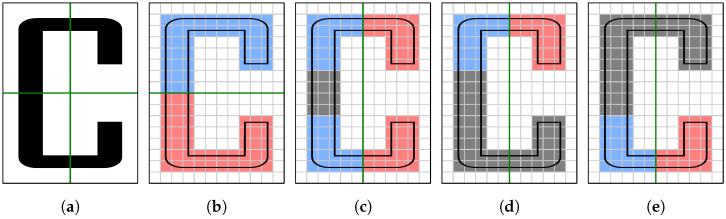
Global, partial, and local reflection symmetry demonstrated on a rasterized letter C: (**a**) input object and its two obvious axes of symmetry (green); (**b**) global symmetry with the horizontal axis splitting the object into two symmetric (blue and red) halves; (**c**) the strongest partial symmetry (next to the global one) and the remaining (gray) object’s pixels; (**d**,**e**) two strongest local symmetries obtained from the considered partial one.

Partial symmetry does not refer to a spatially and/or functionally distinguished part (component) of the system, but to an arbitrary subset of the scene, ranging from a symmetrical pair of two tiny details to the whole scene. On the other hand, local symmetry refers to a spatially distinct symmetrical region that does not contain other (out of the symmetry) points in its interior. Such an area may be, for example, a bounding box, a convex hull, a concave hull or, simply, a connected area, intersecting with a plane of symmetry. Different authors use different informal definitions. We use the last of the above, which could also be called a bilateral partial symmetry. Figure 1 demonstrates the difference between the global, partial, and local symmetries. A 2D input object from Figure 1a has two obvious symmetry axes: the horizontal and the vertical one. The former subdivides the object into two symmetric halves (Figure 1b), representing the global but also the partial and local symmetry. The principle of maximality assures that this is the only partial and local symmetry to be detected with regard to the horizontal axis. The vertical axis defines the maximal partial symmetry from Figure 1c and two maximal local (but not maximal partial) symmetries from Figure 1d,e.

EO data are mostly acquired downwards from satellites, airplanes, drones, etc. This means that the width and length of a considered geographic area are usually much greater than the range of altitudes. Similarly, horizontal distances between in situ environmental sensors are typically longer than the differences of their altitudes. Besides this, many more airborne and satellite EO data samples are collected from the visible top surfaces than from the side and bottom ones. Consequently, it is more likely to explore symmetries from above than from the side. We benefit from this by designing the method to detect only symmetries with nearly vertical symmetry planes. The current implementation reads LiDAR (light detection and ranging) point clouds stored in LAS files only. The symmetries in geometry (shape) are only detected in such data. However, geographical maps are also predominantly two-dimensional, so the above observation can be generalized to symmetry detection in geospatial data other than altitude, which will be explained in more detail in Section 4. Note that the geographical area considered should not be too large anyway, as the method uses Euclidean geometry, ignoring the influence of the Earth’s roundness and cartographic projection.

On account of the previous observation, we had reason to expect significant simplifications and speed-ups of the method, but the next feature of the EO data complicates the method somewhat. Because of the sampling, points of an “original” part and mirrored part rarely match exactly. As a result, the method must detect approximate symmetries instead of ideal exact ones, which is usually a much harder task. This topic is discussed further in Section 2.1.

The bottom-up approach is another crucial feature of the proposed algorithm. By limiting the solution space to vertical symmetry planes only, it suffices to detect symmetries in horizontal slices and then combine them on the basis of the common detected symmetry planes. This constraint crucially contributes to the affordable time complexity of the algorithm in the EO data domain, although the majority of the algorithm’s steps can be easily generalized when we can afford a slower execution. The processing in a single slice can be further decomposed into a search for the so-called basic symmetries, which are merged gradually on the same principles as the symmetries in different slices. The basic symmetry is the symmetry of two geometric primitives, such as points (voxels), line segments, or more complex structures. Our choice is line segments. The core idea is that each line segment which appears in some symmetry should have a symmetric pair (sibling) with the same length somewhere in the slice. The classification of points on the two sides of the symmetry plane is based on a simple vector product test.

A rough outline of the algorithm is presented below, and a more detailed explanation of the individual steps is given in the subsections that follow.

1. Voxelization.
2. Identification (and filtering) of material voxels.
3. Identification of interesting voxels.
4. For each horizontal slice of the voxel grid
  ⊳ Identification of line segments and clustering due to their lengths.
  ⊳ For each cluster of line segments
    * Identification of basic symmetries among pairs of line segments.
    * Merging symmetries.
  ⊳ Merging symmetries from different clusters.
5. Merging symmetries from different slices.
6. For each detected (partial) symmetry *S*
  ⊳ Insertion of “non-interesting” material voxels into *S*.
  ⊳ Extending *S* by mirroring its voxels accurately.
7. Post-processing (eventual detection of global and local symmetries included here).

### 2.1. Voxelization

Voxelization is a space subdivision in all three coordinate directions, resulting in a uniform voxel grid. It is a common acceleration technique in geometric applications, since the arrangement of geometric elements, e.g., points, into the grid cells with constant access time often speeds up various searching operations. However, its main role in the proposed method is in enabling the approximate geometry and, consequently, the approximate symmetry detection. The idea is straightforward: all data samples (e.g., LiDAR points) within the same grid cell (voxel) are approximated with the voxel’s central point. The terms hard or full voxels are sometimes used, as the opposite to air, soft, or empty voxels. The number of the material voxels is usually lower than the number of the input samples, which may accelerate the method significantly. Besides this, the method can be accelerated further, as follows.

Theoretically, the symmetries should be identified among all the material voxels, separately for each horizontal slice of the voxel grid. But this would result in numerous trivial solutions (e.g., “infinitely” many symmetries could be found on a flat surface), so we further reduce the set of candidates for the symmetry detection by extracting the so-called interesting voxels.

**Figure 2:**
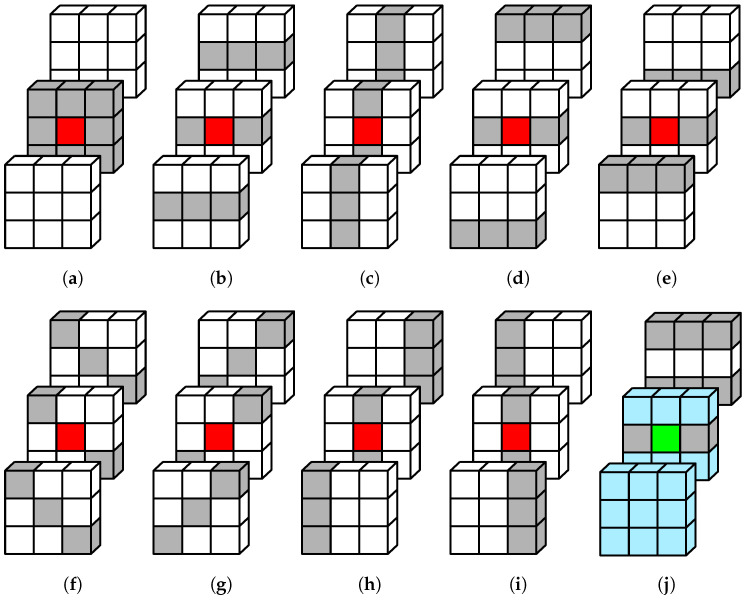
Classification of material voxels according to their surroundings: (**a**–**i**) a voxel on a flat surface of material voxels (colored gray) is uninteresting (colored red) regardless of the status of the other (white) voxels; (**j**) an example of an interesting (green) voxel on a convex edge surrounded by empty (light blue) voxels.

A material voxel is considered interesting for further processing if its 3D surroundings, defined by adjacent material voxels, is not flat. We therefore test each material voxel against the pattern of 26 adjacent voxels and filter out the interesting ones which are not in the middle of a vertical, horizontal, or diagonally slanted local surface. Such interesting voxels represent the input for the next step of line segments identification. Figure 2a–i demonstrates the situations where a central voxel is not identified as interesting. Figure 2j shows an example of the interesting voxel.

The algorithm is fully scalable, as the resolution of voxelization can vary from a few centimeters, or even lower, up to tens or hundreds of meters, depending on the data acquisition technology and the intended use. We currently use a uniform voxel side length in all three coordinate directions. As the EO data are usually georeferenced, the voxel size is entered in meters. Furthermore, the grid coordinate system is oriented such that two axes are horizontal.

### 2.2. Clustering

Voxelization is followed by the identification of line segments, among which the basic symmetries will be searched. A line segment connects the central points of two interesting voxels in the same horizontal voxel grid slice. Furthermore, only line segments, which pass mainly through material voxels, are considered in this step. The basic symmetry detection between two line segments, where one lies on the objects’ surface and the other penetrates the air, is worthless. The default threshold for amount of material voxels in a regular line segment is set to 80%.

**Figure 3:**
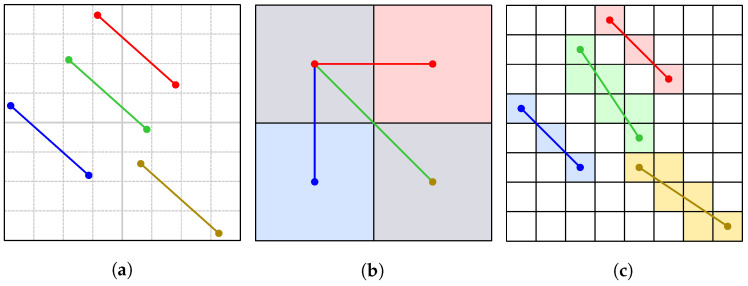
The effect of voxelization on the lengths of line segments and the angles between them: (**a**) four parallel line segments among pairs of input points; (**b**) too large voxels can even cause parallel (blue and red) line segments to become perpendicular or a line segment (yellow) to degenerate into a point; (**c**) for smaller voxels, the deviations and, thus, the tolerances required are significantly smaller.

Two line segments may be symmetric only if they both have the same length. Therefore, the regular line segments are extracted in this step and arranged into clusters due to their lengths. Figure 3 demonstrates the impact of voxelization on the line segments lengths and the angles between pairs of them. Low-resolution voxelization can turn parallel line segments from Figure 3a into perpendicular ones in Figure 3b, or a line segment can even degenerate into a point. Higher-resolution voxelization shown in Figure 3c facilitates this problem. This confirms the meaningfulness of the aforementioned 80% threshold. Besides this, a user defines tolerances for the comparison of lengths and angles in the subsequent step of basic symmetries detection.

### 2.3. Basic Symmetry Detection

**Figure 4:**
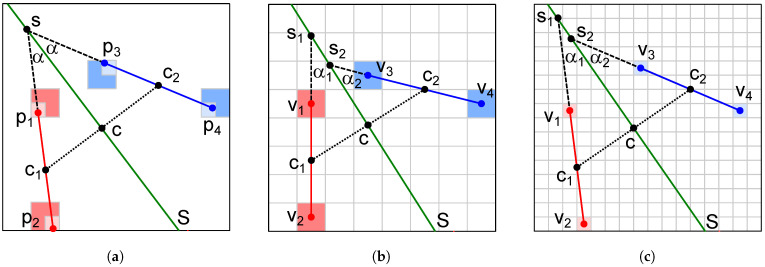
Basic symmetry detection between two line segments: (**a**) exact symmetry between the line segments $p_{1}p_{2}$ and $p_{3}p_{4}$; (**b**) approximate symmetry in a low-resolution voxel space where $p_{1}$, $p_{2}$, $p_{3}$, and $p_{4}$ are replaced by the central points $v_{1}$, $v_{2}$, $v_{3}$, and $v_{4}$ of the corresponding voxels; (**c**) approximate symmetry in a voxel space with higher resolution.

As this step runs separately for each horizontal voxel grid slice, it appears quite straightforward. However, the use of approximate geometry requires a short explanation, which shall be assisted by Figure 4. Note that the situation may be treated as a 2D problem, because the central points of all voxels in an individual horizontal slice have the same *z*-coordinate.

In the ideal geometry from Figure 4a, the test is performed as follows.

1. $c_{1}=\left(p_{1}+p_{2}\right)/2$.
2. $c_{2}=\left(p_{3}+p_{4}\right)/2$.
3. $c=(c_{1}+c_{2})/2$.
4. Determine line *S*, such that: $\left(S⊥\left(c_{1},c_{2}\right)\right)\wedge \left(c\in S\right)$.
5. Determine angle α between $\left(p_{1},p_{2}\right)$ and *S*.
6. If the angle between *S* and $\left(p_{3},p_{4}\right)$ is α, then $p_{1}p_{2}$ and $p_{3}p_{4}$ are symmetric across *S*; otherwise, they are not.

In the approximate geometry from Figure 4b,c, points $p_{1}$ to $p_{4}$ are replaced by the voxel centers $v_{1}$ to $v_{4}$, respectively. The test is then performed as follows.

1. If $\left|\right|v_{1}v_{2}\left|-\right|v_{3}v_{4}\left|\right|>T_{distance}$ then exit without the symmetry detected.
2. $c_{1}=\left(v_{1}+v_{2}\right)/2$.
3. $c_{2}=\left(v_{3}+v_{4}\right)/2$.
4. $c=(c_{1}+c_{2})/2$.
5. Determine line *S*, such that: $\left(S⊥\left(c_{1},c_{2}\right)\right)\wedge \left(c\in S\right)$.
6. Determine angle $\alpha_{1}$ between $\left(v_{1},v_{2}\right)$ and *S*.
7. Determine angle $\alpha_{2}$ between *S* and $\left(v_{3},v_{4}\right)$.
8. If $|\alpha_{1}-\alpha_{2}|\;\leq T_{angle}$, then $v_{1}v_{2}$ and $v_{3}v_{4}$ are symmetric across *S*; otherwise, they are not.

Smaller voxels in Figure 4c allow smaller tolerances $T_{distance}$ and $T_{angle}$ in comparison to Figure 4b, but they significantly increase the time complexity. Note that this is computationally the most demanding task with the theoretical time complexity $O(n^{4})$, where *n* is the number of voxels. This would be achieved if the voxel space had a single slice and each of the $O(n^{2})$ line segments was compared to all $O(n^{2})$ others. The previous steps of extracting interesting voxels and grouping them into clusters not only prevent the calculation of trivial (meaningless) symmetries, but, above all, reduce the number of pairs of line segments that need to be compared here. Note that the theoretical time complexity may differ from the aforementioned $O(n^{4})$ if some other criteria than the altitude is used for the grid slice generation, as explained in Section 4. However, the step of the basic symmetry detection remains the most demanding in any case.

### 2.4. Merging

**Figure 5:**
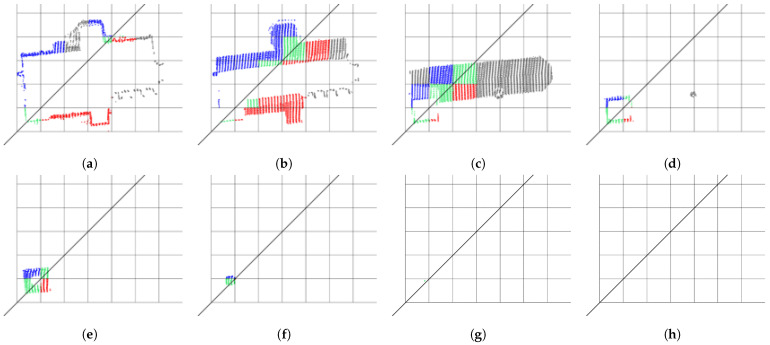
Eight horizontal slices of the grid of 500 voxels, representing the strongest partial symmetry, detected on the Maribor Cathedral. The slices appear in order from: (**a**–**h**) the bottommost to the topmost.

The previous step determines all symmetry planes between the pairs of regular line segments. Next, the algorithm gradually joins all the pairs that share a common plane of symmetry. The thresholds $T_{angle}$ and $T_{distance}$ are reused here to provide approximate plane matching. After the merging is completed in each individual cluster, the process continues to merge the results between the clusters in each individual slice and, finally, between the slices. The black diagonal line in Figure 5 shows the symmetry plane, detected in all eight voxel grid slices, representing the Maribor Cathedral. The slices are arranged from the lowest (Figure 5a) to the highest (Figure 5h). The results obviously allow merging. In this and all subsequent figures, symmetric points on both sides of the symmetry plane are colored red and blue, those in the symmetry plane are green, while the points out of the symmetry are gray. Note that the topmost slice in Figure 5h is not empty, as it contains a point hidden behind the line representing the symmetry plane.

### 2.5. Enhancing the Symmetries by Adding Non-Material Voxels

This subsection addresses both tasks, looped in step 6 of the algorithm, outlined at the end of Section 2:

- Insertion of “non-interesting” material voxels into a symmetry *S*. The interesting voxels only establish symmetries between edges and curved parts of surfaces. This operation pairs the rest of the material (non-interesting) voxels, e.g., those on flat surfaces, with respect to the symmetry plane *S*.
- Extending the symmetry *S* by mirroring its voxels accurately. The previous step mostly raises the density of voxels in *S*. However, in the case where the plane of symmetry is not parallel to one of the horizontal axes of the voxel grid, even this is not sufficient. Let us suppose a pair of symmetric interesting voxels $V_{i}$ and $V_{j}$. Due to the use of the approximate geometry, it may happen easily that some point from $V_{i}$ does not have its pair in $V_{j}$, but in some of its neighboring voxels. If such a voxel is a material one, we add it to *S*. This step significantly improves the quality of the results without spending too much time.

### 2.6. Post-Processing

The result of the previous steps is usually a huge number of detected partial reflection symmetries. A user needs the functionality of navigating through them interactively in a list, sorted from the strongest to the weakest symmetry. The symmetry measure is defined by (1):

(1) $$SM\left(S\right)=\frac{S.voxels}{In.material\_voxels},$$

where S.voxels is the number of voxels in the symmetry *S*, and In.material_voxels is the total number of material voxels, derived from the input set In. Note that 0<SM(S)≤1, where SM(S)=1 corresponds to global symmetries. Besides this sorting, some optional tasks can be performed in the post-processing phase:

- Before sorting, smaller connected parts with too few voxels are filtered out from an individual symmetry. This is actually an iterative process, because the symmetric siblings of the filtered voxels must be removed as well, which can result in new small connected parts. This step is optional, as the part size threshold for filtering can be set to zero voxels. Note that a similar filtering is also performed on the total set of material voxels in the voxelization step.
- Global symmetry detection does not require any special operation, as such symmetries are grouped at the top of the sorted list of partial symmetries. On the other hand, week symmetries with SM(S) below the threshold may be omitted from the bottom of the list.
- Local symmetry detection is run interactively by the user. This functionality is disabled before the partial symmetry detection is completed. Each connected component, intersected by the partial symmetry plane, represents the local symmetry. Optional filtering may be performed then, and a new sorted list is generated afterwards.

## 3. Results

In this section, the algorithm’s performance is demonstrated and analyzed on six different scenes from Slovenia. The tests were run on a personal computer with Intel i9-12900K CPU, 64 GB DDR5 RAM, and Windows 11 Education operating system. The input LAS files were acquired from the LiDAR GIS viewer [45], a publicly available software of the Slovenian Environment Agency. The map of the whole Slovenia is covered by 1 km^2^ tiles, scanned at a resolution of approx. 6 points per m${}^{2}$. Furthermore, LiDAR points in the files were classified as roofs (buildings), trees, and ground (plus unknown), by using the algorithm of Mongus et al. [9,10]. This gave us an opportunity to test the symmetry detection on different layers and their combinations. We did not use the entire 1 km^2^ tiles, but extracted smaller point clouds using the trimming functionality in our proprietary LiDAR data processing software. The considered Slovenian landmarks are displayed in Table 1, while Table 2 lists the parameter values used to control the approximate geometry and the number of accepted solutions.

**Figure 6:**
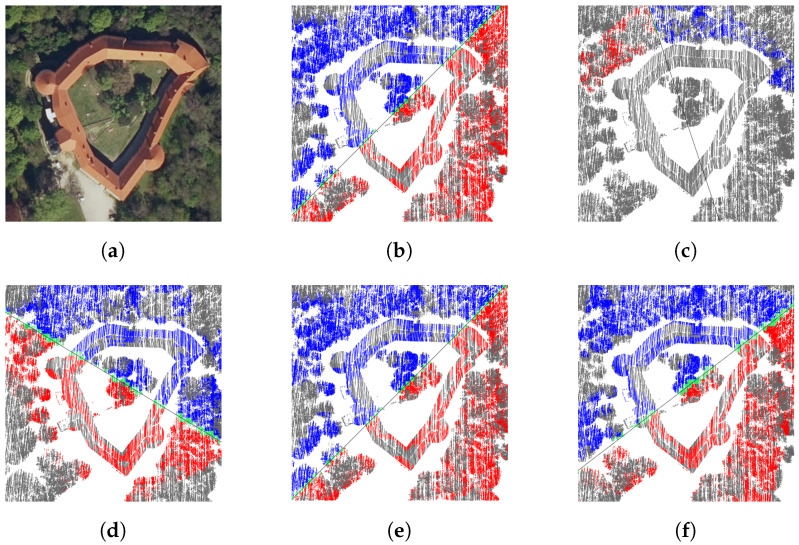
Grad Grad (the Grad castle) test case: (**a**) digital orthophoto; (**b**) strongest partial symmetry on 1 m voxels; (**c**) strongest local symmetry on 1 m voxels; (**d**) 940th strongest partial symmetry on 3 m voxels; (**e**) strongest partial symmetry on 3 m voxels; (**f**) strongest local symmetry on 3 m voxels.

Figure 6 demonstrates the impact of the voxel size on the detected symmetries. The strongest partial symmetries at 1 m and 3 m voxel resolutions (Figure 6b,e, respectively) are practically the same, while the strongest local symmetries (Figure 6c,f) differ significantly. The material voxels at 1 m resolution are distributed more sparsely and, thus, the connected regions are usually smaller. Another interesting observation is that the symmetry in Figure 6d is only at position 940 out of 4438, despite the visually very symmetrical floor plan of the castle with respect to the detected symmetry plane. The reason is that the three towers are all in the same (south, south-west, and west) half of the castle.

**Figure 7:**
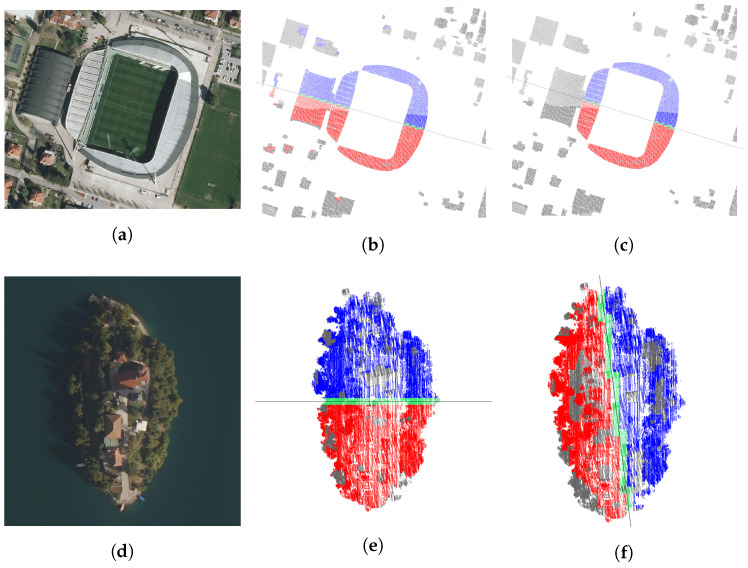

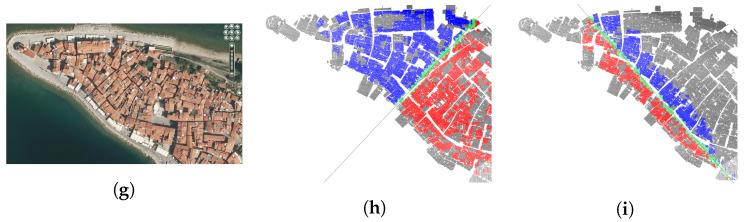
Maribor “Ljudski vrt” stadium, Bled Island, and Punta Piran (Piran cape) test cases. Stadium: (**a**) digital orthophoto; (**b**) strongest partial symmetry; (**c**) strongest local symmetry. Island: (**d**) digital orthophoto; (**e**) strongest partial symmetry; (**f**) strongest local symmetry. Cape: (**g**) digital orthophoto; (**h**) strongest partial symmetry; (**i**) 14,055th strongest partial symmetry.

The results for the stadium (Figure 7a–c) are quite expected. Note that the strongest partial symmetry (Figure 7b) also includes, more or less randomly, some red and blue points on the roofs of the surrounding houses.

The point cloud of Bled Island (Figure 7d–f) was pre-processed to remove the lake surface first. The island has two distinct bilateral symmetries (Figure 7e,f), to which both the ground and the trees strongly conform. Both are so strong that they completely dominate the symmetries of the church and the other buildings on the island.

Punta Piran (Figure 7g–i), the cape of the Piran peninsula, is so densely built up that numerous symmetries are expectedly very strong. The strongest local symmetry is almost identical to the strongest partial symmetry in Figure 7h. The houses rise higher and higher from south-west to north-east, so the plane of the strongest symmetry was correctly expected to run in this direction. Note that the low angle tolerance of 1 degree (see Table 2) results in a large number of very similar symmetries, so that the most symmetrical street on the peninsula represents only the 14,055th strongest local symmetry out of 18,556.

**Figure 8:**
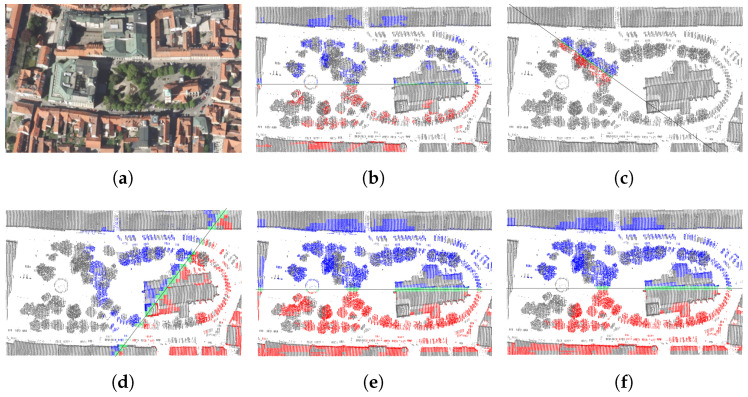
Slomšek square Maribor test case: (**a**) digital orthophoto; (**b**) strongest partial symmetry on 1 m voxels; (**c**) strongest local symmetry on 1 m voxels; (**d**) 2495th strongest partial symmetry on 3 m voxels; (**e**) strongest partial symmetry on 3 m voxels; (**f**) strongest local symmetry on 3 m voxels.

**Figure 9:**
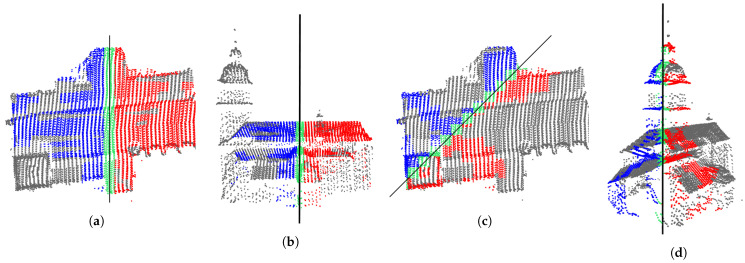
The Maribor Cathedral test case: (**a**) strongest partial symmetry; (**b**) strongest partial symmetry—side view; (**c**) 23rd strongest partial symmetry; (**d**) 23rd strongest partial symmetry—side view.

The results for Slomšek square (Figure 8) are practically the same as those for the Grad castle (Figure 6). The strongest partial symmetries at 1 m and 3 m voxel resolutions (Figure 8b,e, respectively) are practically the same, while the strongest local symmetries (Figure 8c,f) differ significantly. The 2495th strongest partial symmetry out of 5082 in Figure 8d was chosen only, because it coincides with the strongest symmetry of the Maribor Cathedral at a very low resolution (Figure 5). At a slightly higher resolution, this church partial symmetry (Figure 9c,d) is only ranked 23rd out of 64, while the strongest partial symmetry (Figure 9a,b), interestingly, completely ignores the bell tower.

The numerical results from Table 3 were analyzed in two parts. In the first, we compared the figures for the Grad castle in 1 m and 3 m voxel resolutions. In the second part, we tried to draw some conclusions from the results for all six different test sets at 3 m voxel resolution. Although both experiments for the Grad castle started with 273,540 LiDAR points, all other figures are significantly higher at 1 m resolution. The number of voxels is higher by a factor of 26, number of material voxels by a factor of 7.5, and number of interesting voxels by 14. The number of the detected partial symmetries is 7.3 times higher, while the number of local symmetries is higher by factor 55. The latter is not a surprise, as a higher resolution results in worse connectivity of material voxels and, thus, in smaller but more frequent connected parts. As noted during the visual analysis of Figure 6, the strongest partial and local symmetries are nearly the same (SM values 60.47% and 56.58%), while the weakest ones differ significantly (0.91% and 0.01%). Note that, in six of the seven columns, the minimal threshold 5 (see Table 2) for the connected local symmetry regions was reached (row 22 of Table 3). Finally, the processing time at 1 m resolution is 84 times longer than at 3 m resolution (4.5 h against 3 min).

Table 3 displays the results that are understandable to users, and they mostly confirm the expectations. There are, however, some interesting outliers, which would require some additional data to be explained. The processing times (rows 9–11) seem to be correlated with the number of interesting voxels (row 6), as expected, but Punta Piran and Slomšek square are processed significantly slower than the Ljudski vrt stadium. An obvious visual difference between the three cases is that the points (and material voxels) distribution is much more compact in the cases of the square and the cape, and more sparse (distributed into separate groups) for the stadium. This is confirmed with the ratio of the number of local and partial symmetries (rows 8 and 7), which is significantly higher for the stadium. Another observation is that the ratio of the number of interesting voxels and material voxels is higher in urban areas (between 0.51 and 0.57 for the stadium, square, cathedral, and Punta) than in “forests” (0.30 for the castle and 0.31 for the island). However, this last assumption is probably incorrect, because the ratio for the Grad castle in 1 m resolution is 0.58. Eventual answers to these and other interesting questions are further discussed in the concluding Section 4.

## 4. Discussion

In this paper, we presented a new algorithm which detects partial, global, and local reflection symmetries in EO data. To our knowledge, specialized symmetry detection algorithms, designed to benefit from the particularities of EO data, did not exist before, which represented the main research challenge of the presented paper. The nature of these data allowed us to look only for symmetries with near-vertical symmetry planes, but it also made the task more difficult, as we had to design calculations with approximate geometry instead of the much simpler exact geometry. The implemented algorithm was tested and analyzed on LiDAR datasets of six urban and natural attractions in Slovenia of different scales and in different voxel resolutions. It computes thousands of solutions in a few seconds on a usual personal computer. The analysis mostly confirmed the expectations, but there were some interesting outliers, which cannot be explained by observing the inputs and outputs only, but rather require the internal states and data flows to be analyzed as well. In particular, the number of line segments and their distribution into clusters and voxel slices must be studied, together with the distribution of interesting voxels. Overpopulated clusters and slices are assumed to be the main reason for slower performance in some cases. Furthermore, this analysis must be conducted separately for individual data layers and for their different combinations.

Related to the data layers, a new functionality is also planned. It will separately detect symmetries in individual layers (buildings, trees, ground) and then merge them at the topmost merging level. Namely, it usually makes no sense that a voxel on a tree and a voxel on a roof are treated as a symmetric pair.

In a similar manner, the algorithm can be adapted to handle other geospatial data than just bare LiDAR points, where only the geographic coordinates and altitude are considered currently. The prerequisite is that the data are voxelized in 3D or rasterized in 2D. In the case of pure geometric representations, such as irregular triangular networks, where we are not interested in the color or any other measurement or attribute value; this is sufficient. Regardless of the origin of the data, pre-processing may also involve interpolation, decimation, or other ways of changing the resolution. When inserting samples into a grid, it is also important to have in mind that each grid cell will be assigned a single value, e.g., the average of the contained samples. Grayscale and color raster images (satellite imagery, aerial photos, orthophotos, quantized GIS attribute data) may then be processed easily, if colors are interpreted as altitudes. Each color or some range of similar colors then represents a slice. However, a problem is the formation of objects when combining the symmetries of different slices, as the slices often follow each other in a sequence that does not have a proper geometric interpretation. Therefore, the concept of partial symmetry works here without constraints, while the concept of local symmetry is often limited to the adjacency of voxels within a slice. Finally, the approach from the planned tool for handling classified LiDAR data is applicable for values stored in a 3D voxel grid, i.e., the repertoire of values needs to be partitioned into classes that will be handled separately. Let us also note that, when detecting symmetry in non-geometric data, the concept of the interesting voxel may differ from the described one and must be carefully planned.

Another useful functionality, developed already, is an interactive tool that enables a user to interactively enter a reference symmetry plane. The symmetries are then sorted with respect to the similarity of their symmetry planes to the reference one. When a user wants to find out how some particular symmetry is ranged, scrolling through hundreds or thousands of solutions is not of good assistance. The functionality is implemented for both the partial and local symmetry. It was used in this paper already to produce Figure 7i and Figure 8d.

The presented paper does not focus on any specific application of detected symmetries. However, the large number of symmetries found, ordered by the proposed symmetry measure (1), is suitable for further analysis. For example, a locally bounded scene (object) with two distinctly strong symmetries, with orthogonal symmetry planes, is quite likely to be an artificial object, e.g., a building. One can also look for patterns of similar symmetries at different locations (similar objects have similar sets of symmetries). Such combinations can also be used for machine learning.

## Figures and Tables

**Table 1:** Selected testing landmarks in Slovenia.

Landmark	Part of Slovenia	GPS (N, E)	Area [m × m]	Figure
Grad castle	North-East	(46.800, 16.096)	140 × 140	Figure 6
Ljudski vrt	North-East	46.563, 15.640)	280 × 240	Figure 7a–c
Bled Island	North-West	(46.362, 14.090)	150 × 200	Figure 7d–f
Punta Piran	South-West	(45.529, 13.567)	400 × 300	Figure 7g–i
Slomšek square	North-East	(46.559, 15.645)	240 × 160	Figure 8
Maribor Cathedral	North-East	(46.559, 15.645)	80 × 50	Figure 5 and Figure 9

**Table 2:** Test parameters.

Parameter	Value
Min. number of interesting voxels in connected region (pre-processing filter)	5
Min. number of voxels in connected symmetrical region (post-processing filter)	5
Distance tolerance [m]	0.1
Angle tolerance [deg.]	1
Min. length of line segment [voxel]	4
Min. connected region in local symmetry [voxel]	5

**Table 3:** Settings and results for six test cases in Slovenia.

Row	Measure	Grad Castle	Grad Castle	Ljudski Vrt	Slomšek Square	Maribor Cathedral	Bled Island	Punta Piran
1	Layers ^1^	B+T	B+T	B	B+T	B	B+T+G	B
2	Points	273,540	273,540	126,714	88,107	11,779	219,186	99,413
3	Voxel size [m]	1	3	3	3	3	3	3
4	Voxels	1,629,909	62,169	98,496	42,480	5415	9520	25,326
5	Material voxels	89,851	12,190	4507	4864	704	1738	3114
6	Interesting voxels	52,142	3721	2579	2478	380	538	1699
7	Partial symmetries	32,263	4438	6863	5082	64	979	12,177
8	Local symmetries	476,393	8616	24,404	15,629	73	1030	18,556
9	Time [s] for partial symmetries	15,741	182	23	35	0.03	2.38	44
10	Time [s] for local symmetries	287	9	2	4	0.01	0.3	6
11	Total time [s]	16,028	191	25	39	0.04	2.68	50
12	Points in strongest partial symmetry	75,182	166,968	68,393	45,359	7626	189,632	63,851
13	Voxels in strongest partial symmetry	23,597	7372	2050	2675	361	1412	1798
14	% of voxels in strongest partial symmetry	26.26	60.47	45.48	54.99	51.27	81.24	57.73
15	Points in weakest partial symmetry	24	2703	796	1273	1194	2810	440
16	Voxels in weakest partial symmetry	7	112	19	31	66	1 38	16
17	% of voxels in weakest partial symmetry	0.01	0.91	0.42	0.63	9.37	2.18	0.51
18	Points in strongest local symmetry	23,353	154,958	49,191	42,393	7415	189,403	60,229
19	Voxels in strongest local symmetry	7386	6898	1346	2488	349	1405	1691
20	% of voxels in strongest local symmetry	8.22	56.58	29.86	51.15	49.57	80.84	54.3
21	Points in weakest local symmetry	6	14	69	140	21	148	115
22	Voxels in weakest local symmetry	5	5	5	5	7	5	5
23	% of voxels in weakest local symmetry	0.01	0.04	0.11	0.1	0.99	0.28	0.16

^1^ B—buildings, G—ground, T—trees.

## Data Availability

The source code of the symmetry detection software is available at https://github.com/luckyLukac/ReflectionSymmetryDetection, accessed on 18 July 2023. LAS tiles of the entire Slovenian territory are publicly accessible at http://gis.arso.gov.si/evode/profile.aspx?id=atlas_voda_Lidar@Arso&culture=en-US, accessed on 18 July 2023. For information about the trimmed datasets used in the experiments, software for clipping LAS files, etc., please contact the author L.L. by email.

## Abbreviations

The following abbreviations are used in this manuscript:

DEM	Digital elevation model
EO	Earth observation
GIS	Geographic information system
GPS	Global Positioning System
LAS	LAS (LASer) File Format
LiDAR	Light detection and ranging
OECD	The Organisation for Economic Co-operation and Development
PCA	Primary component analysis
SM	Symmetry measure
